# Involvement of occupational physicians in the management of MRSA-colonised healthcare workers in Germany – a survey

**DOI:** 10.1186/1745-6673-8-16

**Published:** 2013-05-27

**Authors:** Madeleine Dulon, Frank Haamann, Albert Nienhaus

**Affiliations:** 1 Institution for Statutory Accident Insurance and Prevention in the Health and Welfare Services, Hamburg, Germany; 2 University Medical Center Hamburg-Eppendorf, Institute for Health Services Research in Dermatology and Nursing, Martinistrasse 52, Hamburg, 20246, Germany

**Keywords:** Methicillin-resistant Staphylococcus aureus, Colonisation, Persistent carriage, Healthcare worker, Occupational disease

## Abstract

**Background:**

Colonisation of healthcare workers (HCWs) with methicillin-resistant *Staphylococcus aureus* strains (MRSA) is a challenge for any healthcare facility. Persistent carriage of MRSA among HCWs causes special problems, particularly in occupational-medical care. German occupational physicians responsible for healthcare facilities were therefore asked about their experience in managing MRSA-colonised HCWs.

**Methods:**

In May 2012, 549 occupational physicians were asked in writing about in-house management of MRSA-colonised HCWs. The semi-standardised survey form contained questions about collaboration between the infection control team and the occupational physician, the involvement of the occupational physician in in-house management of MRSA carriers and the number of persistently colonised HCWs in 2011. The answers were intended to apply to the largest facility cared for by the occupational physician.

**Results:**

207 occupational physicians took part in the survey (response rate 38%). In 2011, 73 (35%) occupational physicians were responsible for the occupational-medical management of an average of four MRSA-colonised HCWs. Eleven doctors (5.3% of 207) managed a total of 17 persistently colonised HCWs. One of these 17 employees was dismissed. In the case of MRSA carriage among HCWs, most occupational physicians cooperated with the infection control team (77%) and 39% of occupational physicians were responsible for the occupational-medical management of the affected carrier. 65% of facilities had specified policies for the management of MRSA-colonised HCWs. After the first MRSA-positive screening result, 79% of facilities attempt to decolonise the affected employee. In 6% of facilities, the colonised HCWs were excluded from work while receiving decolonisation treatment. In 54% of facilities, infection control policies demand the removal of MRSA carriers from patient care.

**Conclusions:**

Not all facilities have policies for the management of MRSA-colonised HCWs and there are major differences in occupational consequences for the affected HCWs. In order to protect both the employees and the patients, standards for the in-house management of MRSA colonisation in HCWs should be developed.

## Background

In recent reviews, the prevalence of methicillin-resistant *Staphylococcus aureus* (MRSA) in healthcare workers (HCWs) was estimated to be about 5% [1,2]. MRSA carriage of HCWs was higher in the endemic setting (8.1%) than during outbreaks (3.9%) [1]. The main reservoir of MRSA is the anterior nares. Outside the nose, MRSA is found at many other body sites (e.g. hands, throat, perineum, chronic skin lesions) and additionally at sites of invasive procedures [3]. MRSA carriage may be intermittent or persistent. This distinction is important because persistent carriage is associated with a higher organism burden than intermittent carriage, resulting in an increased risk of acquiring infections [4,5]. Intermittent carriage is often self-limiting and requires no special treatment in healthy people [6] unless preventive skin decolonisation is indicated in order to reduce the risk of developing invasive infections [7].

The main route of transmission of MRSA is considered to be from patient to patient via the contaminated hands of HCWs [1,2]. Staphylococcal hand carriage in HCWs is usually transient – detectable after a shift and gone before the next shift [1]. Still, HCWs have been implicated as the source in a number of published outbreak reports [1,8]. Particularly HCWs with skin lesions or upper respiratory tract infections – associated with higher rates of persistent colonisation – are considered to be at risk of transmitting MRSA to patients [1]. Such events are rare [9] and the relative importance of the colonised staff in the transmission of MRSA in the endemic setting is unclear [1,2]. This might explain why the recommended infection control measures in national guidelines are controversial [1,7].

German guidelines recommend the decolonisation of a colonised healthcare worker and removal of the affected employee from patient care until proven eradication [10-12]. There is general uncertainty about the optimal policy [13] and practical issues, such as management of persistently colonised HCWs, are unresolved questions [1,7]. There is also the fact that compensation for preventive decolonisation measures in HCWs is neither provided by statutory health insurance nor by statutory accident insurance. Therefore, with respect to stigmatisation and occupational consequences for persistently colonised HCWs, a solution in insurance law has been demanded [13,14].

When caring for MRSA-colonised HCWs, occupational medicine and infection control are subject to two different and potentially contradictory legal regulations in Germany. In order to protect employees working with biological substances, the German Ordinance on Biological Agents (BioStoffV) regulates occupational healthcare which must be provided by the occupational physician who is responsible for the affected facility [15]. In order to protect patients from nosocomial infections, the German Infection Protection Act (IfSG) regulates the prophylactic measures of the spread of resistant pathogens within the medical facility [16]. Infection control practitioners are responsible for implementing this law. Nowhere is it explicitly specified that these two professional groups should work together in the practical management of colonised HCWs in the health service.

The purpose of this paper is to provide information on the kind of policies that exist for the management of MRSA-colonised HCWs and to investigate the extent to which occupational physicians are involved in the occupational-medical care of MRSA carriers in general and in particular of persistently colonised HCWs.

## Methods

In May 2012, 549 occupational physicians were asked in writing about in-house management of MRSA-colonised HCWs. The occupational physicians were identified from a data base relating to addresses of participants invited to training courses, organised from the German Social Accident Insurance Institution for the Health and Welfare Services (BGW), which is the workers’ compensation board for all non-public healthcare and welfare providers. Only those physicians responsible for occupational-medical care in healthcare facilities were chosen. The standardised questionnaire was administered to solicit information about the involvement of occupational physicians in in-house management of MRSA carriers, occupational-medical care of MRSA carriers and the numbers of persistent carriers which were cared for by the occupational physician. The answers were intended to apply to the year 2011 and the largest facility cared for by the occupational physician. The survey was carried out anonymously and study participation was voluntarily. Data processing was in accordance with the German Data Protection Act. To increase the response rate, three skin care cases (“Hautschutzkoffer”) were raffled for use in the occupational health practice. The analysis was descriptive. Data are presented as count (percentages).

## Results

Of the eligible participants, 207 (38%) of 549 occupational physicians chose to enrol in the survey. The physicians were responsible for various areas in the health service, including facilities for clinical and nursing care, homes for the handicapped and physiotherapy clinics. Regulations for the management of MRSA-colonised HCWs existed in 65% of the facilities, specified as a component of an infection control protocol or as part of the employment agreement (Table 1).

**Table 1:** Involvement of occupational physicians in the in-house management of MRSA-colonised healthcare workers (n=207)

**Statement**	**Responses**	**N**	**%**
Regulations for the management of MRSA-colonised employees exist	As a component of an infection control protocol	123	59
	As part of an employment agreement	12	6
	Not established	72	35
Collaboration takes place between occupational physician and infection control staff with respect to MRSA-colonised employees	Yes	155	77
	No	52	23
Responsibility for the coordination of the eradication therapy is regulated	Yes	128	62
	No	79	38
The occupational physician is informed of positive MRSA findings	Always	83	42
	Predominantly	47	24
	Seldom/never	77	34
The occupational physician is responsible for dealing with MRSA-colonised employees	Yes	88	39
	No	119	61
The occupational physician is responsible for screening employees	Always	8	4
	Predominantly	23	11
	Seldom/never	176	85

After a positive MRSA finding in a healthcare worker, there was collaboration between the occupational physician and the responsible infection control practitioners in 77% of the facilities. 62% of the occupational physicians declared that the responsibility for coordinating eradication therapy had been clarified in the facility they were responsible for. In 36% of cases, the occupational physician was responsible for coordination of the measures of the eradication therapy, either alone or in collaboration with the infection control professional, the attending physician or the nursing service (no table). 42% of the occupational physicians considered that they were always informed of positive MRSA findings in HCWs. 39% of the occupational physicians were responsible for occupational-medical care of MRSA carriers, including notification and counselling. 15% of the occupational physicians were responsible for screening procedures.

In 2011, 73 occupational physicians (35%) managed a mean of four employees colonised with MRSA. The affected employees predominantly worked in hospitals and nursing homes and more rarely in homes for the handicapped or in an outpatient service (Table 2). A few were working in physiotherapy or veterinary clinics.

**Table 2:** Areas of work in which MRSA-colonised healthcare workers were managed by occupational physicians in 2011 (n=73)

**Area of work**	**N**^**1**^
Hospital, clinic, convalescence clinic	54
Nursing home	26
Home for the handicapped	10
Outpatient service	8
Other (physiotherapy, veterinary clinic, ambulance service)	4

^1^ Multiple answers possible.

In the case of MRSA-positive screening swabs, affected HCWs were forced to undergo decolonisation therapy in 79% of the facilities after the first positive screening result (Table 3). In 7% of the facilities, decolonisation was only recommended when the HCWs exhibited risk factors, such as a weakened immune system, a wish for children, symptoms of discomfort or if they worked with patients at risk.

**Table 3:** Regulations on the decolonisation of healthcare workers after MRSA- positive screening results; answers given by occupational physicians (n=207)

**Regulations on decolonisation**	**N**	**%**
After first positive finding	163	79
If the healthcare worker displays risk factors^1^	14	7
Unknown	30	14

^1^ Immune deficiency, a wish for children, symptoms of discomfort, contact with patients at risk.

Forty-four occupational physicians (21%) had to evaluate the success of decolonisation treatment by follow-up cultures in the year 2011 (no table). Follow-up cultures were taken by 66% (29/44) on three consecutive days, starting three days after completion of treatment measures. Additional follow-up cultures after one and three months were taken by eleven physicians.

In 70% of the facilities, the MRSA-colonised HCWs were subject to work restrictions, although there were differences in the extent of the restrictions (Table 4). In 6% of the facilities, colonised HCWs were forced to take leave of absence until documentation of negative follow-up cultures was obtained. In almost half the facilities (54%), colonised HCWs were able to continue work, but not with patient contact or in high-risk units, nor if they suffered from a chronic skin disease. In 10% of the facilities, colonised HCWs were allowed to continue work without restrictions other than compliance with hand hygiene measures and use of protective equipment during high-risk activities.

**Table 4:** Work restrictions for MRSA-colonised healthcare workers to continue their work; answers given by occupational physicians (n=205)

**Work restrictions**	**N**	**%**
1 (= compliance with hand hygiene and use of protective equipment^1^)	20	10
1 + No patient contact	28	14
1 + No work in high-risk units^2^	31	15
1 + No patient contact, no work in high-risk units^2^	32	15
1 + No chronic skin diseases, no patient contact, no work in high-risk units^2^	21	10
Forced leave of absence as long as screening results are positive	12	6
Unknown/not fixed	61	30

^1^ Gloves, protective clothing, disposable aprons and a face mask for high-risk activities.

^2^ Units containing vulnerable patients at high risk of developing invasive infection.

In 2011, eleven occupational physicians managed a total of 17 HCWs who were persistently colonised with MRSA. In one case, the employee was dismissed as a consequence of the persistent colonisation, presumably for health reasons.

## Discussion

Colonised HCWs are generally asymptomatic, but they are likely to be important in the transmission of MRSA, most frequently acting as vectors [1]. If HCWs are still colonised with MRSA after several attempts at decolonisation, it may not be possible for them to continue their professional work. HCWs have good reason to be worried about the potential occupational consequences of an MRSA colonisation, as has been reported by several authors [8,13,14,17] and is evident from the employee who was dismissed. Our survey showed that there are major differences between healthcare facilities with respect to management of MRSA-colonised HCWs. The policies range from unrestricted continued work with adherence to basic infection control guidelines, to continued work with major or minor restrictions in the activities or area of work, to forced leave of absence.

The question of whether and for how long colonised HCWs should be excluded from work is unresolved [1,2,7]. Official German recommendations lay down that MRSA carriers can resume patient contact once they have had MRSA-negative swabs on three consecutive days, starting three days after completion of the eradication treatment [10]. However, this specific topic is given a low level of evidence [10], as the effectiveness of different screening strategies for considering staff (or patients) free from MRSA carriage remains controversial [1,2,7]. In a few German studies, clinical infection control professionals have reported that they do not require MRSA-colonised HCWs to be removed from patient care, provided that the affected HCWs comply with basic infection control measures [18,19]. However, these in-house regulations did not apply during outbreaks or when colonised HCWs were suffering from chronic skin diseases [18] or were working in high-risk units [19]. German recommendations for facilities outside the clinical setting, for nursing homes or domestic care, consider that adherence to the basic principles of hand hygiene is the most important measure and allow colonised HCWs to continue to work [20]. This recommendation is restricted to non-outbreak situations and is being justified with the reduced risk of transmitting resistant strains in long-term care facilities [21].

About 80% of the occupational physicians cooperated with infection control practitioners with respect to the management of MRSA-colonised HCWs. As a limitation of our study, the quality and extent of this collaboration and the point of view of the infection control practitioners were not assessed. However, only 42% of occupational physicians stated that they always felt adequately informed about MRSA findings in employees. In general, it was striking that 30% of occupational physicians failed to provide information about work restrictions and 14% did not know the regulation scheme for the decolonisation in the facility they were responsible for. This lack of information may either be due to the lack of binding regulations within the facility, or may be taken as a sign that the occupational physician is not adequately involved in the in-house management of MRSA carriers. The reason that occupational physicians are not involved in staff screening, is probably that staff screening in Germany is only advocated in outbreaks [10] and the control measures during outbreaks are the responsibility of the infection control experts [16].

Nevertheless, close collaboration between the occupational physician and infection control practitioners is essential in managing an MRSA-colonised employee, as both the employee and the patients must be protected [12]. Guidelines for this collaboration are given by the “consensus recommendation Baden-Wuerttemberg”, which was developed by infection control professionals, public health experts and occupational physicians [22]. The occupational physician can serve as a case manager for the MRSA-colonised HCW. However, in order to be able to create a confidential relationship with the healthcare worker afflicted, anonymity of this employee should be ensured and an agreement between management and representatives of the workers concerning social safety of the employee afflicted is needed.

In the present study, a total of 17 persistently colonised HCWs were treated by occupational physicians in the course of 2011. However, this number cannot be used for an estimation of the prevalence of persistently colonised MRSA carriers among German HCWs because of missing denominator data and the method of recording used in our study. In all but one of these 17 cases, persistent carriage was established on the basis of two or more unsuccessful eradications. Although there is no general consensus on how to define persistent carriage of MRSA, repeated unsuccessful eradication seems to be the current standard in clinical practice [22].

The diversity and uncertainty regarding the occupational-medical care of persistent MRSA colonisation is partly caused by missing regulations regarding the coverage of the cost involved. In Germany, infections associated with MRSA can be recognised as occupational disease and the medical as well as the occupational and social rehabilitation cost can be covered by the workers’ compensation board [23,24]. So far, MRSA colonisation is not considered an occupational disease and the compensation board is therefore not able to cover the cost for testing and treating afflicted HCWs. However, persistent colonisation with MRSA is associated with an increase in the risk of infection [25,26]. Therefore decolonisation of HCWs with persistent colonisation not only reduces nosocomial transmission, but will reduce infection risk for the HCW and therefore avoids the development of an occupational disease as well. As it is the task of the compensation board to avoid the development of a looming occupational disease, the cost of decolonisation can be covered by the compensation board [27].

In 2011, 73 occupational physicians (35% of all responders) were responsible for an average of four HCWs colonised with MRSA. However, these numbers do not allow for the estimation of the prevalence of MRSA colonisation of HCWs in Germany. First of all, the response rate of our survey was low (38%), which hampers the transferability of our study results. As occupational physicians who had experience with MRSA might have been more likely to participate in the survey, the number of colonised HCWs might be overestimated. Secondly, the involvement of occupational physicians in the management of MRSA was quite diverse and often non-existent. As our only source of information was the occupational physicians, this might have introduced an underestimation of the number of HCWs colonised.

## Conclusion

Even though some recommendations exist, the management of HCWs with MRSA colonisation is not standardised in Germany. Important questions in this realm remain unsolved, e.g. which tasks can be performed by colonised HCWs, when should preventive decolonisation treatment be performed, who should be responsible for the management of MRSA-colonised HCWs, and who covers the cost involved? Answering these questions is key to the development and implementation of standards for the management of MRSA-colonised HCWs.

## Competing interests

The authors declare that they have no competing interests.

## Authors’ contributions

MD participated in the design of the study, performed the statistical analyses, interpreted the data and drafted the manuscript. FH participated in the design of the study, interpreted the data and drafted the manuscript. AN interpreted the data and drafted the manuscript. All authors read and approved the manuscript.
